# Whether Green Finance Can Effectively Moderate the Green Technology Innovation Effect of Heterogeneous Environmental Regulation

**DOI:** 10.3390/ijerph19063646

**Published:** 2022-03-18

**Authors:** Yong Fang, Zhenquan Shao

**Affiliations:** School of Economics and Management, Beijing University of Chemical Technology, Beijing 100029, China

**Keywords:** green finance, environmental regulation, green technology innovation, moderating effect, spatial spillover effect

## Abstract

As an essential way to promote ecological civilization, green finance is attracting wide attention. However, whether green finance can successfully regulate the green technology innovation effect of heterogeneous environmental regulations and boost green technology innovation in coordination with heterogeneous environmental regulations remains unclear. Based on the re-measurement of the green finance development index of various provinces and cities in China, this study uses the spatial Durbin model to test the above problems empirically. The results show that green finance and “market incentive” environmental regulations can promote regional green technology innovation, while “command and control” environmental regulations inhibit regional green technology innovation. Green finance plays a negative regulatory role in the mechanism of heterogeneous environmental regulations affecting green technology innovation. Green finance alleviates the negative impact of “command and control” environmental regulations on green technology innovation and weakens the positive impact of “market-incentive” environmental regulations on green technology innovation. In terms of spillover effects, green finance can effectively promote green technology innovation in neighboring regions, while heterogeneous environmental regulations have a crowding-out effect on green technology innovation in neighboring regions.

## 1. Introduction

Since its reform and opening-up, China has achieved an economic miracle of rapid growth over a long period of time. However, with the continuous advancement of industrialization and urbanization, the extensive economic growth model with high investment and high energy consumption is emerging as a culprit in resource shortages and environmental pollution. The sustainable development of the economy and society is facing severe challenges. To address the unfavorable situation of economic growth at the expense of the environment, the Central Committee of the Communist Party of China put forward the strategic deployment of promoting green development and accelerated the reform of the ecological civilization system in the report of the 19th National Congress of the Communist Party of China [1]. As a fundamental way to drive green development, not only will green technology innovation promote the rapid development of regional economies, but it will also urge enterprises to strengthen the research and development of green technology and green processes to contribute to the balance between economic growth and environmental protection [2,3]. However, green technology innovation has “dual externalities” of technology and the environment. This strong externality leads to a decrease in the efficiency of social resource allocation, which should be avoided through environmental regulation policies [4].

The existing discussions on environmental regulation and green technology innovation can be categorized into two types: the crowding out effect and the innovation compensation effect. The crowding out theory holds that environmental regulation forces enterprises to internalize environmental costs, allocating corresponding funds for pollution prevention and control. When the capital quota is limited, this impact will occupy the share of capital that enterprises use for green technology innovation, and increase the financial pressure on enterprises [5,6,7,8]. Suppose it is difficult for enterprises to invest significant money into researching and developing green technology. In that case, they will take negative measures instead, such as moving enterprises from areas with high environmental regulation intensity to areas with low environmental regulation intensity, which will affect the vitality of regional green technology innovation [9,10]. The innovation compensation effect theory takes Porter’s hypothesis as the premise and argues that environmental regulation, as an incentive factor for green technology innovation, can effectively promote the diffusion of green technology innovation [4]. To help enterprises meet pollution emission standards and alleviate the financial pressure caused by green technology innovation activities, the government strengthens intellectual property protection of green technology and gives certain tax incentives. While realizing value creation, enterprises that meet pollution emission standards have incentive and demonstration effects on other enterprises, thus promoting regional green technology innovation [11,12]. In addition, some scholars believe that the impact of environmental regulation on green technology innovation is uncertain [13,14]. It is not difficult to find that scholars have not reached a consistent conclusion on the relationship between environmental regulation and green technology innovation. At the peak of the growth of green technology innovation activities in China, it is of great significance to promote ecological civilization, including understanding of the role of environmental regulation and the facilitation of regional pollution control based on green technology innovation.

As an important starting point to promoting the sustainable development of regional economies and the environment, green finance aims to provide market-oriented capital guarantees for green technologies, green projects, and green industries through capital allocation. In recent years, green finance has received extensive attention from academia, the research scope of which mainly focuses on the economic benefits of green industries [15,16,17] and the environmental benefits of green projects [18,19,20]. As an important method of alleviating enterprises’ constraints, innovative financing can provide continuous financial support for green technology innovation, and is an important force for promoting green technology innovation. However, only a few studies have touched on the influence of green finance on green technology innovation. Therefore, it is important to investigate whether green finance plays a role in promoting green technology innovation and what role it plays, in order to support current management practices and theoretical research. In addition, considering the multidimensional and complex nature of China’s environmental policies, it is difficult to ensure the implementation process of regional green technology innovation by relying on a single environmental policy. Only mutually coordinated and supportive environmental policies can effectively promote regional green development. In comparison, the existing research does not explore the collaborative mechanism of green finance and environmental regulation. Accordingly, this article is an important supplement to the existing research that analyzes whether green finance can effectively alleviate the financial pressure brought by the current environmental regulation, and promote green technology innovation activities in coordination with environmental regulation.

This paper examines how the implementation of green finance and environmental regulations influences green technology innovation from the perspective of regional environmental governance. The contributions of this study are mainly focused on the following two aspects. The first is the incorporation of green finance, environmental regulation, and green technology innovation into the same research framework for the first time. Previous studies have only separately discussed the impact of green finance or environmental regulation on green technology innovation. On this basis, this paper further explores the synergistic effect of green finance and environmental regulation, and the moderating effect of green finance in the mechanism of heterogeneous environmental regulation on green technology innovation, which effectively reveals the internal relationship between environmental regulation, green finance, and green technology innovation. Second, considering the regional correlation among green finance, environmental regulation, and green technology innovation, this study uses the spatial econometric model for experimental design, which avoids the errors in experimental results caused by traditional econometric models. Based on the spatial perspective, the study provides a beneficial expansion for guiding green finance and environmental regulation policies to coordinate regional governance.

This paper is organized as follows. Section 2 is the theoretical analysis and research assumptions of green finance, environmental regulation, and green technology innovation. Section 3 introduces the model, data source, and measurement method for variables. Section 4 analyzes and tests the empirical results. Apart from concluding the research, Section 5 also provides policy recommendations, and points out the limitations of the research and future research directions.

## 2. Theoretical Basis and Research Hypothesis

### 2.1. Environmental Regulation and Green Technology Innovation

As the environment belongs to the public good, enterprises are not willing to take the initiative to bear the cost of its protection. In this case, if the government does not formulate rules to maintain public goods, what emerges will be the disorder of free-market. Therefore, there is a reliable theoretical basis for the government to formulate environmental regulations. The environmental regulation policies of government intervention are mainly divided into two categories: “command and control” and “market incentives” [21]. “Command and control” environmental regulation refers to the government formulating different types of laws, regulations, or standards to regulate enterprise behavior. Its purpose is to force enterprises to comply with corresponding environmental regulations through strict pollution index limits, guidelines, and corresponding penalties [22]. Such environmental regulations are widely used all over the world; the “Environmental Protection Law of People’s Republic of China”, the “Law of the People’s Republic of China on the Prevention and Control of Atmospheric Pollution”, and the “Law of the People’s Republic of China on the Prevention and Control of Water Pollution” all belong to this category of measures. Whether “command and control” environmental regulation can promote green technology innovation mainly depends on the standards set by the government. The government often adopts a “one size fits all” management mode, resulting in not only a lack of incentive for environmental protection enterprises to carry out green innovation, but also a low probability of polluting enterprises carrying out technology innovation due to capital constraints. “Command and control” environmental regulation rarely generates a good incentive effect. Sometimes, it even initiates the opposite effect [23,24]. “Market incentive” environmental regulation refers to the incorporation of market factors into regulatory policies. In general, the government relies on market means to guide the pollutant discharge behavior of enterprises, increasing the costs of enterprises through the implementation of sewage charging systems, discharge permit systems, taxation, deposit return systems, policy subsidies, and other systems, and compelling enterprises to carry out pollution control and technology innovation [22]. This kind of environmental regulation involves the “polluter pays principle”, which can objectively reduce the R&D risk expectation of enterprises aimed at reducing pollution output, improve the income expectation of enterprise R&D investment, and enhance the willingness of enterprises to adopt environmental R&D to improve green technology [25,26]. Accordingly, this research proposes the following hypotheses:

**Hypothesis 1a** **(H1a).** *“Command and control” environmental regulation has a significant positive impact on regional green technology innovation.*

**Hypothesis 1b** **(H1b).** *“Market incentive” environmental regulation has a significant positive impact on regional green technology innovation.*

### 2.2. Green Finance and Green Technology Innovation

As an important means pf balancing economic and environmental benefits, green finance is an important extension of traditional financial tools in the environmental field. Financial institutions are believed to fully consider the costs and benefits related to environmental factors in the process of capital allocation, thereby promoting sustainable social development [27]. Specifically, green finance can internalize the negative externalities generated by corporate pollution emissions and dynamically adjust the opportunity cost of environmental pollution through capital allocation to both increase green investment and reduce pollution investment, thus realizing the purpose of using capital allocation to guide the green transformation of industrial structures. On the one hand, for polluting enterprises, the development mode of green finance restrict them to a certain extent [28]. By raising the loan interest rate of polluting enterprises to increase debt financing costs, green finance can not only effectively restrain polluting investment expenditures, but also force enterprises to carry out green technology innovation activities [29]. On the other hand, the development mode of green finance will grant environmental protection enterprises with availability and convenience in obtaining financing, thereby guiding more social funds to the green environmental protection industry. Green finance alleviates the financing pressure of environmental protection enterprises by increasing the loan amount and reducing the loan interest rate [30], enhancing the willingness of environmental protection enterprises to carry out green innovation activities and effectively expediting the sustainable development of the green industry [31,32]. Therefore, this research proposes the following hypothesis:

**Hypothesis 2** **(H2).** *Green finance has a significant positive impact on regional green technology innovation.*

### 2.3. Green Finance, Environmental Regulation, and Green Technology Innovation

Under “command and control” environmental regulation, the “one size fits all” management mode adopted by government departments leads to the lack of a driving force of green innovation for both environmental protection enterprises and polluting enterprises [24]. However, green finance can play a positive moderating effect on the influence mechanism of “command and control” environmental regulation on green technology innovation [33]. Green finance can effectively improve the green innovation ability of environmental protection enterprises by providing them with a large amount of financial support in enhancing the risk-resisting ability. For polluting enterprises, green finance further increases the production cost of enterprises by raising the financing threshold through compulsory purchase of green insurance and carbon emission rights trading, forcing them to reduce costs through green technology innovation and thereby improving the green technology innovation willingness of polluting enterprises. At this stage, China’s “market incentive” environmental regulation has been relatively complete, and the relevant collection standards have approached the cost of pollution control. Under “market incentive” environmental regulation, environmental protection enterprises tend to carry out green innovation activities rather than paying pollution discharge fees. Therefore, the promoting effect of financial support of green finance is relatively limited. While polluting enterprises need to pay high pollution discharge fees under “market incentive” environmental regulation, their working capital is occupied in large quantities. In response to national policies, green finance further reduces investment in polluting enterprises and aggravates the tension of working capital of polluting enterprises, thus hindering green technology innovation [34]. Therefore, green finance cannot synergize with “market incentive” environmental regulation to promote green technology innovation. Based on this, this research proposes the following hypotheses:

**Hypothesis 3a** **(H3a).** *Green finance plays a negative regulatory role in the impact mechanism of “command-and-control” environmental regulation on green technology innovation. Green finance and “command-and-control” environmental regulation synergistically promote regional green technology innovation.*

**Hypothesis 3b** **(H3b).** *Green finance plays a negative regulatory role in the impact mechanism of “market incentive” environmental regulation on green technology innovation. Green finance and “market incentive” environmental regulation cannot synergistically promote regional green technology innovation.*

### 2.4. The Spatial Effects of Green Finance and Environmental Regulations

Differences in geographical distribution lead to a strong spatial correlation in the behaviors of local governments, producing spatial spillover effects. As an important means of regional environmental governance, green finance and environmental regulation will not only incentivize local enterprises to carry out green technology innovation, but will also have a certain radiating effect on the surrounding areas. There are three main mechanisms for the spatial spillover of green finance and environmental regulation on green technology innovation. Firstly, there is a strategic interaction between green finance and environmental regulation policies among local governments [35], that is, the implementation intensity of local green finance and environmental regulation policies will affect the intensity of green finance and environmental regulation policies in surrounding areas, thereby affecting green technology innovation in surrounding areas. Secondly, green technology innovation has a spatial spillover effect [36], which means that the impact of local green finance and environmental regulation policies on green technology innovation will affect the level of green technology innovation in surrounding areas through the spillover of green technology. Thirdly, due to differences in the implementation intensity of green finance and environmental regulation policies in different regions, the flow of production factors between regions may occur, leading to the migration of enterprises that cannot adapt to the intensity of the policy, thus producing negative spatial spillover effects on adjacent regions. Accordingly, this study puts forward the following assumptions:

**Hypothesis 4** **(H4).** *“Command and control” environmental regulation and “market incentive” environmental regulation have significant spatial spillover effects on green technology innovation.*

**Hypothesis 5** **(H5).** *Green finance has significant spatial spillover effects on green technology innovation.*

**Figure 1:**
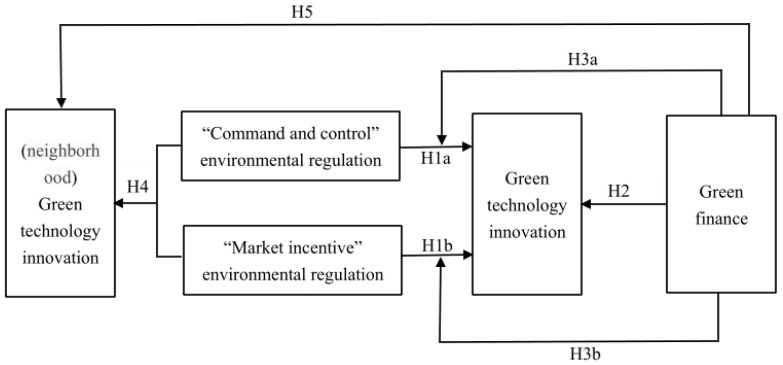
Conceptual framework.

In summary, the theoretical and mechanistic analyses of the hypotheses between green finance, environmental regulation, and green technology innovation are shown in Figure 1.

## 3. Data and Methodology

### 3.1. Econometric Model

Regarding the close regional correlation among environmental regulation, green finance, and green technology innovation, to better reflect the spatial effect of economic variables and to avoid the estimation bias of traditional econometric models when there is a spatial correlation, a spatial econometric model is used to examine the impact of environmental regulation and green finance on green technology innovation in the spatial category. With the preliminary establishment of a spatial autoregressive model (SAR), spatial errors model (SEM), and spatial Durbin model (SDM), this paper selects the optimal model for subsequent analysis through various tests. The effects of heterogeneous environmental regulation and green finance on green technology innovation is examined so as to verify the research hypotheses H1a, H1b, and H2. The specific model settings are as follows:

SAR:

(1) $$gti_{i,t}=\beta_{0}+\rho Wgti_{i,t}+\beta_{1}gf_{i,t}+\beta_{2}er_{1_{i,t}}+\beta_{3}Z_{i,t}+\varepsilon_{i,t}$$

(2) $$gti_{i,t}=\beta_{0}+\rho Wgti_{i,t}+\beta_{1}gf_{i,t}+\beta_{2}er_{2_{i,t}}+\beta_{3}Z_{i,t}+\varepsilon_{i,t}$$

SEM:

(3) $$gti_{i,t}=\beta_{0}+\beta_{1}gf_{i,t}+\beta_{2}er_{1_{i,t}}+\beta_{3}Z_{i,t}+\gamma_{i,t},\;\;\gamma_{i,t}=\lambda W\mu_{i,t}+\varepsilon_{i,t}$$

(4) $$gti_{i,t}=\beta_{0}+\beta_{1}gf_{i,t}+\beta_{2}er_{2_{i,t}}+\beta_{3}Z_{i,t}+\gamma_{i,t},\;\;\gamma_{i,t}=\lambda W\mu_{i,t}+\varepsilon_{i,t}$$

SDM:

(5) $$gti_{i,t}=\beta_{0}+\rho Wgti_{i,t}+\beta_{1}gf_{i,t}+\beta_{2}er_{1_{i,t}}+\beta_{3}Z_{i,t}+\theta_{1}Wgf_{i,t}+\theta_{2}Wer_{1_{i,t}}+\theta_{3}WZ_{i,t}+\varepsilon_{i,t}$$

(6) $$gti_{i,t}=\beta_{0}+\rho Wgti_{i,t}+\beta_{1}gf_{i,t}+\beta_{2}er_{2_{i,t}}+\beta_{3}Z_{i,t}+\theta_{1}Wgf_{i,t}+\theta_{2}Wer_{2_{i,t}}+\theta_{3}WZ_{i,t}+\varepsilon_{i,t}$$

where subscripts *i* and *t* indicate the province and period, respectively; gti represents explained variable regional green technology innovation; gf represents the level of green finance development; $er_{1}$ and $er_{2}$ represent “command and control” environmental regulation and “market incentive” environmental regulation, respectively; *Z* represents a vector of the control variables, which includes the degree of investment openness (IO), the level of urbanization (UR), the degree of trade openness (TO), the human capital of environmental protection system (HC) and intellectual property protection (IPP); *W* represents the spatial weight matrix; ρ represents the spatial autoregressive coefficient; λ represents the spatial error term coefficient; $\beta_{0}$ represents the constant term; $\beta_{1}$, $\beta_{2}$, and $\beta_{3}$ represent the regression coefficient of the level of green finance development, heterogeneous environmental regulation, and control variables, respectively; $\theta_{1}$, $\theta_{2}$ and $\theta_{3}$ represent the spatial regression coefficient of the level of green finance development, heterogeneous environmental regulation and control variables, respectively; ε represents the error term; γ and μ represent the random disturbance term.

To further explore the effect of the joint implementation of heterogeneous environmental regulation and green finance and whether green finance has a moderating effect on the green innovation effect of heterogeneous environmental regulation, the research hypotheses H3a and H3b are verified. After taking into account existing research, this paper first subtracts the sample mean and then adds the interaction term of green finance and environmental regulation into the model [37]. The specific settings of the model are as follows:

SAR:

(7) $$gti_{i,t}=\beta_{0}+\rho Wgti_{i,t}+\beta_{1}gf_{i,t}+\beta_{2}er_{1_{i,t}}+\beta_{3}Z_{i,t}+\beta_{4}\left(er_{1_{i,t}}-\bar{er_{1_{i,t}}}\right)\times \left(gf_{i,t}-\bar{gf_{i,t}}\right)+\varepsilon_{i,t}$$

(8) $$gti_{i,t}=\beta_{0}+\rho Wgti_{i,t}+\beta_{1}gf_{i,t}+\beta_{2}er_{2_{i,t}}+\beta_{3}Z_{i,t}+\beta_{4}\left(er_{2_{i,t}}-\bar{er_{2_{i,t}}}\right)\times \left(gf_{i,t}-\bar{gf_{i,t}}\right)+\varepsilon_{i,t}$$

SEM:

(9) $$gti_{i,t}\;=\;\beta_{0}\;+\;\beta_{1}gf_{i,t}\;+\;\beta_{2}er_{1_{i,t}}\;+\;\beta_{3}Z_{i,t}\;+\;\beta_{4}\left(er_{1_{i,t}}\;-\;\bar{er_{1_{i,t}}}\right)\;\times \;\left(gf_{i,t}\;-\;\bar{gf_{i,t}}\right)\;+\;\gamma_{i,t,},\;\;\gamma_{i,t}\;=\;\lambda W\mu_{i,t}\;+\;\varepsilon_{i,t}$$

(10) $$gti_{i,t}\;=\;\beta_{0}\;+\;\beta_{1}gf_{i,t}\;+\;\beta_{2}er_{2_{i,t}}\;+\;\beta_{3}Z_{i,t}\;+\;\beta_{4}\left(er_{2_{i,t}}\;-\;\bar{er_{2_{i,t}}}\right)\;\times \;\left(gf_{i,t}\;-\;\bar{gf_{i,t}}\right)\;+\;\gamma_{i,t,},\;\;\gamma_{i,t}\;=\;\lambda W\mu_{i,t}\;+\;\varepsilon_{i,t}$$

SDM:

(11) $$\begin{array}{cc} gti_{i,t}= & \beta_{0}+\rho Wgti_{i,t}+\beta_{1}gf_{i,t}+\beta_{2}er_{1_{i,t}}+\beta_{3}Z_{i,t}+\beta_{4}\left(er_{1_{i,t}}-\bar{er_{1_{i,t}}}\right)\times \left(gf_{i,t}-\bar{gf_{i,t}}\right)\\ \; & +\theta_{1}Wgf_{i,t}+\theta_{2}Wer_{1_{i,t}}+\theta_{3}WZ_{i,t}+\theta_{4}\left(er_{1_{i,t}}-\bar{er_{1_{i,t}}}\right)\times \left(gf_{i,t}-\bar{gf_{i,t}}\right)+\varepsilon_{i,t} \end{array}$$

(12) $$\begin{array}{cc} gti_{i,t}= & \beta_{0}+\rho Wgti_{i,t}+\beta_{1}gf_{i,t}+\beta_{2}er_{2_{i,t}}+\beta_{3}Z_{i,t}+\beta_{4}\left(er_{1_{i,t}}-\bar{er_{1_{i,t}}}\right)\times \left(gf_{i,t}-\bar{gf_{i,t}}\right)\\ \; & +\theta_{1}Wgf_{i,t}+\theta_{2}Wer_{2_{i,t}}+\theta_{3}WZ_{i,t}+\theta_{4}\left(er_{2_{i,t}}-\bar{er_{2_{i,t}}}\right)\times \left(gf_{i,t}-\bar{gf_{i,t}}\right)+\varepsilon_{i,t} \end{array}$$

where $\bar{gf}$, $\bar{er_{1}}$, and $\bar{er_{2}}$ represent the means of the level of green finance development, “command and control” environmental regulation, and “market incentive” environmental regulation, respectively; $\beta_{4}$ and $\theta_{4}$ represent the regression coefficient and spatial regression coefficient of the interaction term between green finance and environmental regulation after subtracting the sample mean.

### 3.2. Direct, Indirect, and Total Effects

In order to explore the mechanism of green finance and environmental regulation on green technology innovation while observing the specific effects on the region and neighboring regions, it is necessary to decompose the spillover effect of the spatial model. According to the different scopes and objects of spatial effects, LeSage and Pace [38] separate the effects of independent variables on dependent variables in spatial econometric models into total effects, direct effects, and indirect effects. The total effect reflects the average impact of the independent variable on all regions, the direct effect reflects the average impact of the independent variable on the dependent variable in the region, and the indirect effect reflects the average impact of the independent variable on the dependent variable in other regions. Furthermore, LeSage and Pace find that the utilization of partial differential methods can explain the effects of variable changes in different model settings [39], which provides an essential methodological basis for validating the research hypotheses H4 and H5. As shown in Equation (13), a decomposition of the spatial overflow effect is proposed.

(13) $$Y={\left(1-\rho W\right)}^{-1}+{\left(1-\rho W\right)}^{-1}\left(X\beta +WX\theta \right)+{\left(1-\rho W\right)}^{-1}\varepsilon$$

Therefore, the direct effect can be measured using the arithmetic method of the elements on the main diagonal, and the indirect effect can be measured with the arithmetic method of the elements off the diagonal.

### 3.3. Spatial Weight Matrix

The existing literature is mostly based on the spatial adjacency weight matrix and geographic distance weight matrix expansion. As the stock of R&D capital is a key factor in promoting green technology innovation, it is of great significance to examine the spatial dependence of R&D capital. Therefore, based on the traditional space matrix, this paper constructs an R&D distance weight matrix to conduct an exploratory analysis of the efficiency of green technology innovation. The specific spatial weight matrix is set as follows:

The geographical distance weight matrix $\left(w_{1}\right)$ is set with the reciprocal of the latitude and longitude distance between the two provincial capitals as the weight. The form is as follows: $w_{ij}=1/d_{ij}$, i≠j; $w_{ij}=0$, i=j.

The spatial adjacency weight matrix $\left(w_{2}\right)$ is set based on whether the geographic locations of the two provinces are adjacent or not. The adjacent value is 1 and the non-adjacent value is 0. The form is as follows: $w_{ij}=1$, i≠j; $w_{ij}=0$, i=j.

The R&D distance weight matrix $\left(w_{3}\right)$ takes the absolute value of the reciprocal of the R&D capital stock distance as the weight setting and the form is as follows: $w_{ij}=1/R$&D, i≠j; $w_{ij}=0$, i=j. The calculation method of R&D capital stock is based on the research ideas of Zhang et al. [40].

### 3.4. The Test of Spatial Autocorrelation

The spatial correlation of variables relies on the premise of employing the spatial econometric model. In this paper, Moran’s I is used to measure the spatial correlation of variable. The calculating formula is as follows:

(14) $$\mathrm{Moran}’s\;I=\frac{\sum_{i=1}^{n}\sum_{j=1}^{n}W_{ij}\left(Y_{i}-\bar{Y}\right)\left(Y_{j}-\bar{Y}\right)}{S^{2}\sum_{i=1}^{n}\sum_{j=1}^{n}W_{ij}}$$

where $S^{2}=\frac{1}{n}\sum_{i=1}^{n}\left(Y_{i}-\bar{Y}\right)$ and $\bar{Y}=\frac{1}{n}\sum_{i=1}^{n}Y_{i}$. $Y_{i}$ and $Y_{j}$ represent the number of green patents of province *i* and province *j*, respectively. $\bar{Y}$ represents the mean number of green patents, *n* represents the total number of provinces examined, and $W_{ij}$ is the spatial weight matrix. The value of Moran’s I is between −1 and 1, with a positive Moran’s I indicating a positive correlation of green technology innovation, and a negative value indicating a negative correlation of green technology innovation.

### 3.5. Variable Description and Data Source

We use panel data from 30 provinces in China from 2010 to 2017. Tibet, Macao, Hong Kong, and Taiwan are excluded because of the unavailability of relevant data. The data comes from the *China Environmental Yearbook*, the *China Industrial Economic Statistical Yearbook*, the *China Science and Technology Statistical Yearbook*, the *China Insurance Yearbook*, the *China City Statistical Yearbook*, the *Banking Social Responsibility Report*, the *State Intellectual Property Office*, and the *Tonghuashun iFinD database*. The value method and symbol of related variables are as follows.

#### 3.5.1. Explained Variable

Green Technology Innovation (gti): The green patents not only effectively reflect the innovation and application of green technologies such as resource conservation and environmental protection, but also intuitively reflect the level and scale of regional green innovation. Using the research of Dong et al. as reference [41], the State Intellectual Property Office was searched by international patent classification number according to the definition of green technology patent proposed by the World Intellectual Property Organization. The logarithm of green patent applications was used to characterize the level of regional green technology innovation.

#### 3.5.2. Core Explanatory Variables

Green Finance Development (gf): According to the *Guiding Opinions on Building a Green Financial System* issued in 2016, the green financial system includes four parts: green credit, green securities, green insurance, and green investment. Green credit is represented by the balance of energy-saving and environmental protection loans and the interest expenditures of high-energy-consuming industries of industrial enterprises above designated sizes. The energy-saving and environmental protection loan balance is obtained from the *Banking Social Responsibility Report*, which has only national-level data. Therefore, by using the ideas for measuring private capital of Li and Wei [42], this study further assumes that each proportion of regional energy conservation and environmental protection loans to the national energy conservation and environmental protection credit scale is the same as the proportion of various regional financial institutions in the national financial institution loans, which is used to calculate the balance of energy conservation and environmental protection loans in each region. Green securities are expressed by the market value of A-share environmental protection companies and high-energy-consuming industrial companies. Green insurance is expressed by the agricultural insurance expenditure of each province, and green investment is expressed by investment in industrial pollution control and financial expenditures for energy conservation and the environmental protection industry. This research uses the entropy method to measure the development level of green finance based on the determination of each indicator layer and the calculation ideas of Yang and Sun [43].

Environmental regulation: This research divides environmental regulation tools into “command and control” environmental regulation $\left(er_{1}\right)$ and “market incentive” $\left(er_{2}\right)$ environmental regulation. The former compels enterprises to comply with relevant regulations by setting environmental standards and market access thresholds; the latter regulates the output benefits of enterprises through market means, such as the collection of sewage charges.

“Command and control” environmental regulation $\left(er_{1}\right)$: in accordance with the research methods of Ye et al. [44], the comprehensive emission index of pollutants (industrial wastewater, industrial SO${}_{2}$, and industrial smoke) per unit output value is measured to evaluate the intensity of “command control” environmental regulation.

Firstly, standardize the above three types of pollutant indicators:

(15) $$UE_{i,j}^{s}=\left[UE_{i,j}-\min\left(UE_{j}\right)\right]/\left[\max\left(UE_{j}\right)-\min\left(UE_{j}\right)\right]$$

where $UE_{i,j}$ is the index value of class *j* pollutants in province *i*, $\min(UE_{j})$ and $\max(UE_{j})$ are the minimum and the maximum value of the class *j* pollutants, and $UE_{i,j}^{s}$ is the standardized value of the class *j* pollutants in province *i*.

Secondly, calculate the adjustment coefficient of each pollutant index. The formula is as follows:

(16) $$W_{j}=UE_{i,j}/\bar{UE_{i,j}}$$

where $UE_{i,j}$ is the provincial average level of emission per unit output value of the class *j* pollutant.

Finally, calculate the comprehensive index of environmental regulation of each province $\left(ER_{i}\right)$:

(17) $$ER_{i}=\frac{1}{3}\sum_{j=1}^{3}W_{j}UE_{i,j}^{s}$$

“Market incentive” environmental regulation $\left(er_{2}\right)$: According to Zhu et al. [45], the logarithm of the amount of pollution discharge fees in each province represents the “market incentive” environmental regulation method.

#### 3.5.3. Control Variables

With the assistance of existing research, this study selected investment openness, urbanization, trade openness, the human capital in the environmental protection system, and intellectual property protection as the control variables. Among them, the degree of investment openness (IO) is the proportion of foreign direct investment in each region to the gross product, the level of urbanization (UR) is the proportion of the urban population in each region to the total population, the degree of trade openness (TO) is the ratio of the total trade volume of each region to the total output value, human capital of environmental protection system (HC) is the proportion of the number of environmental protection system personnel in each region to the total population, and intellectual property protection (IPP) refers to the percentage of regional technology market transactions in the total output value. The descriptive statistical results of the above variables are shown in Table 1.

## 4. Empirical Results and Analysis

### 4.1. Results of Spatial Autocorrelation Test

Clarifying the spatial correlation degree of green technology innovation is a prerequisite for analyzing the spatial effects of green finance, environmental regulation, and the combination of the two. Mainly, Moran’s I is adopted to test the spatial correlation of green technology innovation. As shown in Table 2, under the R&D distance weight matrix, the positive spatial autocorrelation coefficients of green technology innovation in various regions of China from 2010 to 2017 are significant, indicating a clustering effect in green technology innovation in various regions of the country. This is because the stickiness of green innovation knowledge (especially tacit knowledge) and innovative technology depends on the spatial distance between the two parties of knowledge transfer [46]. Enterprises in the same area are more likely to transfer knowledge between different knowledge potentials due to their similarity of technology and location.

### 4.2. Model Selection

In order to choose a spatial econometric model suitable for this study, Elhorst’s idea was referred to in order to conduct the Lagrange Multiplier test and Robust Lagrange Multiplier test [47]. The Lagrange Multiplier-error test (LM-error test) tests the applicability of the spatial error model (SEM), the Lagrange Multiplier-lag test (LM-lag test) tests the applicability of the spatial autoregressive model (SAR), and the Robust Lagrange Multiplier-error test (Robust LM-error test) and the Robust Lagrange Multiplier-lag test (Robust LM-lag test) are stability supplements to the Lagrange multiplier test. If the LM-error is found to be more significant than the LM-lag in the test, the Robust LM-error is significant and the Robust LM-lag is not significant; then the SEM model suitability can be determined theoretically. It can be seen from the test results in Table 3 that under the geographical distance weight matrix, the *p*-value of the LM-error and Robust LM-error tests of the model is less than 0.1. In other words, the null hypothesis will be rejected at the significance level of 10%, apart from which the LM-lag and Robust LM-lag tests do not reject the null hypothesis. Theoretically, the SEM model should be used. Next, the Hausman test of three spatial measurement models (as shown in Table 4) is carried out, which shows that random effects are more appropriate than fixed effects. Finally, this paper compares the SEM and SDM models based on the actual regression results. The regression results of Models (4) to (9) under the geographical distance weight matrix ($w_{1}$) are shown in Table 5.

-value of the LM-error and Robust LM-error tests of the $er_{1}$ model is less than 0.1, that is, the null hypothesis is rejected at the significance level of 10%, and the LM-lag and Robust LM-lag tests do not reject the null hypothesis. In theory, the SEM model should be used. Secondly, the Hausman test of three spatial measurement models (as shown in Table 4) is carried out, and the results show that random effects are more appropriate than fixed effects. Finally, this paper compares the SEM model and the SDM model based on the actual regression results. The regression results of models (4) to (9) under the geographical distance weight matrix $\left(w_{1}\right)$ are shown in Table 5.

In Table 5, Columns (1), (3), (5), and (7) are the regression results without interactive items, and Columns (2), (4), (6), and (8) are the regression results with interactive items. As can be observed, the SDM model and SEM model can well reflect the spatial effect of green technology innovation under the geographical distance weight matrix. The core explanatory variables and control variables have good significance in the model with interactive terms, and the fitting degree is also close. In the SDM model, the spatial lag of green finance, environmental regulation, and the interaction terms of the two plays a significant role under the geographical distance weight matrix; that is, the influence of the spatial lag on the model construction cannot be neglected. Therefore, this study uses the treatment methods of You and OuYang [48] to select the spatial econometric model, which suggests that the SDM model will eliminate the errors caused by missing variables without causing more errors in the empirical results. Therefore, it is reasonable to use the SDM model for follow-up research.

### 4.3. Results of Spatial Models

On the whole, provincial green technology innovation under the geographical distance weight matrix, the spatial adjacency weight matrix, and the R&D distance weight matrix has significant spatial effects, indicating that green technology innovation in this province will significantly impact green technology innovation in other provinces. Owing to the good significance levels of the coefficients in Columns (1) and (2) and Columns (7) and (8), the regression results under the geographical distance weight matrix are selected for specific analysis.

Models (1) and (7) in Table 6 are regression results without interaction terms. The regression coefficient of “command and control” environmental regulation is evidently negative, indicating that “command and control” environmental regulation ($er_{1}$) has a significant inhibitory effect on regional green technology innovation. This is inconsistent with the analysis results of Chen et al. [49], which we believe is induced by the different research periods of the articles. Because “command and control” environmental regulation generally imposes mandatory requirements on enterprises through policies, regulations, orders, and other means, in the short term, enterprises can be promoted to reform through mandatory means to achieve compliance standards. By contrast, in long-term reform and adjustment, enterprises are no longer constrained by mandatory regulatory policies, which may even have a particular inhibitory effect on enterprise green technology innovation. Therefore, the research hypothesis H1a holds.

Differing from the “command and control” environmental regulation, the regression coefficient of the “market incentive” environmental regulation($er_{2}$) is significantly positive, indicating that the “market incentive” environmental regulation has a significant role to play in promoting green technology innovation. This is consistent with the analysis results of Zhang et al. [50]. As a matter of fact, many scholars have found that the effect of “market incentive” environmental regulation on green technology innovation is better than that of “command and control” environmental regulation [51,52]. This is because “market incentive” environmental regulations generally use methods such as pollution discharge fees and environmental taxes to force enterprises to carry out technology innovation and environmental governance. At this stage, China’s “market incentive” environmental regulations are relatively mature, and the relevant collection standards and the impact on enterprises have approached or exceeded the benefits brought by enterprises’ pollutant discharges. Considering the long-term interests, enterprises are more inclined to increase technology investment in green innovation and reduce costs through technological progress to obtain long-term benefits. Therefore, research hypothesis H1b holds.

The regression coefficient of green finance (gf) is significantly positive, indicating that green finance can effectively promote regional green technology innovation, consistent with the views of Yu et al. [32], Zhang et al. [53], and Hu et al. [54]. As an important method of regional environmental governance, green finance can effectively curb polluting investment spending and force polluting enterprises to engage in green technology innovation activities by raising loan interest rates and lowering loan limits. Furthermore, it can make financing for environmental enterprises easier by lowering loan interest rates and increasing loan limits to guide social capital to the environmental industry; as a result, enterprises become more willing to practice green innovation. Therefore, hypothesis H2 holds.

Models (2) and (8) in Table 6 are regression results that include interaction terms. The coefficients of the interaction terms between “command and control” environmental regulations and green finance are evidently positive, indicating that green finance alleviates the negative impact of “command and control” environmental regulation on green technology innovation, and promotes regional green technology innovation in coordination with “command and control” environmental regulation. On the one hand, green finance can provide environmental protection enterprises with a large amount of financial support. They have more capital for trial and error during research and development, effectively lowering their tendency to avoid risks and thus enhancing green technology innovation. On the other hand, green finance has a significant financing penalty effect and investment inhibition effect on polluting enterprises. It exacerbates financial pressure on polluting enterprises and forces them to carry out green technology innovation by raising the financing threshold and forcing them to buy green insurance and other financial methods. Therefore, green finance alleviates the negative impact of “command and control” environmental regulation on green technology innovation and achieves the benign interaction. Hence, the research hypothesis H3a holds. The coefficients of the interaction terms between “market incentive” environmental regulation and green finance are significantly negative, indicating that the current combination of green finance development and “market incentive” environmental regulation has significantly inhibited the development of green technology innovation. This implies that China’s current green finance negatively affects the impact mechanism of “market incentive” environmental regulation on green technology innovation. It weakens the positive impact of “market incentive” environmental regulation on green technology innovation. Hence, the research hypothesis H3b holds. This is because under the “market incentive” environmental regulation, environmental protection enterprises tend to carry out green innovation activities rather than paying high pollution charges; therefore, the effect of green finance on environmental protection enterprises’ willingness to innovate is minimal. Simultaneously, polluting enterprises need to pay high sewage fees, and green finance exacerbates polluting enterprises’ working capital. Accordingly, this may cause polluting enterprises to migrate from places with high pollution discharge fees to places with low pollution discharge fees, ultimately hindering polluting enterprises’ green innovation behavior.

From the perspective of control variables, the level of urbanization (UR), the degree of trade openness (TO), and the level of intellectual property protection (IPP) have a significant role in promoting regional green technology innovation. In comparison, the level of human capital in the environmental protection system (HC) is not effective in improving the level of regional green technology innovation, and investment openness (IO) only plays a significant role in promoting Models (6) to (10).

### 4.4. Spillover Effect of Green Technology Innovation

Spatial spillover effects can effectively measure the effect of explanatory variables on the explained variables in related regions. The total effect is the numerical sum of the direct and the indirect effect. The direct effect reflects the degree of influence of the explanatory variable on the local area, and the indirect effect reflects the degree of influence of the explanatory variable on neighboring areas. The spatial spillover effects of the er1 model and the er2 model under the geographical distance weight matrix are shown in Table 7. “Command and control” environmental regulations have a significant inhibitory effect on green technology innovation in local and neighboring regions. The “market incentive” environmental regulations have promoted green technology innovation in the region while restraining green technology innovation in neighboring regions. Research hypothesis H4 is therefore verified. The technical standards set by “command and control” environmental regulations should correspond to the country’s development level. Considering the fact that there is still a certain gap between China’s technological level and that of developed countries, high technical standards set in the region may cause local enterprises to become inert in green technology innovation, resulting in a decline in the level of local green technology innovation. Meanwhile, the linkage effect of inter-regional policies will raise the regulatory standards of neighboring regions, causing negative spillover of green technology innovation to neighboring regions. Under ’market-incentivized’ environmental regulation, the high level of environmental regulation in the region attracts the gathering of environmental-friendly enterprises, which will increase the level of green technology innovation in the region, while differences in the implementation of policies between neighboring regions cause the migration of polluting enterprises to regions with less stringent environmental regulations, resulting in negative spillover effects in neighboring regions.

Green finance has significant direct and indirect effects on green technology innovation, indicating that the development of green finance can not only significantly improve the level of green technology innovation in the region, but can also promote the effective development of green technology innovation in neighboring areas through spatial spillover effects. Therefore, hypothesis H5 is verified. This is because green finance provides important financial support for technology innovation and the reformation of local enterprises. For environmental protection enterprises with abundant funds, green finance can increase capital allocation, awarding them with more trial and error opportunities in green technology innovations. For environmental protection enterprises with tight funds, green finance alleviates their financing pressure and increases their investment in green technology innovation. Due to the technological spillover effect, high-level environmental protection enterprises in this region may adopt technology transfer and transactions to adjacent areas to increase enterprise income, with a promoting effect on adjacent areas.

The direct effects of the interaction between “command and control” environmental regulation and green finance, and the interaction term between “market incentive” environmental regulation and green finance are significant. Nevertheless, the indirect and total effects are not meaningful, indicating that the mutual influence of the two is only effective for local green technology innovation, but not for neighboring regions. This is because there is a lack of a coordinated mechanisms between governments when making environmental policies, and governments only pay attention to the influence of the implementation of policies on their regions.

## 5. Conclusions and Policy Recommendations

### 5.1. Conclusions

Green technology innovation is the cornerstone of promoting regional sustainable development, and green finance and environmental regulation are important forces guiding regional green technology innovation. This study aims to investigate the effect of green finance and environmental regulation on green technology innovation, and further explore the synergistic implementation effect of green finance and environmental regulation and the moderating effect of green finance on heterogeneous environmental regulation’s green technology innovation effect. In consideration of the spatial correlation among green finance, environmental regulation, and green technology innovation, this study uses the spatial Durbin model to analyze the above problems. The following conclusions were obtained:

First, regional green technology innovation has a significant spatial agglomeration effect; green finance can effectively promote the improvement of regional green technology innovation; “command and control” environmental regulations significantly inhibit regional green technology innovation, while “market incentive” environmental regulations effectively promote regional green technological innovation.

Second, green finance plays a negative regulatory role in the mechanism of heterogeneous environmental regulations on green technology innovation. The difference is that green finance alleviates the negative impact of “command and control” environmental regulations on green technological innovation and weakens the positive impact of “market incentive” environmental regulations on green technological innovation.

Third, in terms of spatial spillover effects, “command and control” environmental regulations have a significant inhibitory effect on local and neighboring green technology innovations; “market incentive” environmental regulations can promote local green technology innovation efficiency, but with an innovation crowding-out effect on neighboring regions. Not only can green finance significantly improve the level of local green technology innovation, but it can also promote the effective development of green technology innovation in neighboring areas through space overflow.

### 5.2. Policy Recommendations

First, improve the green financial system, explore and innovate green financial products, and further increase the role of green finance in promoting regional green technological innovation. Although the scale of green finance in China is currently in the leading position globally, it is still lower than that of traditional finance. Therefore, it is difficult to support regional industrial technological progress and green transformation and upgrading. It is necessary to further increase the investment scale of green finance, enrich the types of green finance products, and encourage it to play more influential role in green technology innovation.

Second, formulate environmental regulation policies that are coordinated with the development of green finance, and give play to the synergistic effect of green finance and environmental regulation on regional green technology innovation. At present, China is facing severe environmental problems. For sustainable development, formulating environmental regulatory policies of appropriate intensity is indispensable. The combination of green finance and environmental regulations can effectively alleviate the financial pressure of enterprises due to technological upgrades and improve their innovative technology level, thus achieving a “win-win” between economic development and environmental protection.

Third, strengthen information exchange among regional governments and formulate green finance and environmental regulation policies for cross-regional cooperation. Insufficient communication among regional governments will reduce the efficiency of green technology innovation among regions. Building a high-level dialogue mechanism among local governments, breaking administrative and technical barriers, overall planning of green finance and environmental regulation policies, and systematic cross-regional cooperation can expand green technology. The diffusion effect of financial and environmental regulations, in turn, promotes the free flow of green innovation factors among regions.

### 5.3. Research Deficiencies and Prospects

The remaining limitations of this study are as follows. First, due to the availability of data, the time range of this study is from 2010 to 2017, which lacks the value of reference and comparison in the time dimension, and it would be better to extend the research period by updating and adjusting the core indicators. Second, this study does not divide China into different regions for further exploration. Given that different regions have notable differences in economy, culture, and institutions, this may have a certain impact on the empirical results. In follow-up research, we will effectively divide the regions and examine differences in experimental results caused by regional heterogeneity. Third, in terms of selecting control variables, what has been considered in this paper may not have been comprehensive, and there are still other factors that affect green technology innovation. Subsequent research will enrich and improve the selection of control variables.

## Figures and Tables

**Table 1:** Descriptive statistics of the variables.

Variable	Observation	Mean	Standard Deviation	Minimum	Maximum
*gti*	240	7.7905	1.3811	3.6889	10.8129
*gf*	240	0.1163	0.0683	0.0371	0.4171
*er* ${}_{1}$	240	0.1313	0.1834	0.0000	1.1714
*er* ${}_{2}$	240	8.6575	1.5700	4.6205	11.8889
IO	240	0.0226	0.0181	0.0001	0.1138
UR	240	0.5599	0.1268	0.2575	0.8960
TO	240	0.2905	0.3291	0.0168	1.5482
HC	240	0.0002	0.0001	0.0001	0.0003
IPP	240	0.0119	0.0255	0.0002	0.1602

**Table 2:** Moran’s I of green technology innovation in 30 provincial administrative regions from 2010 to 2017.

Year	$w_{1}$			$w_{2}$			$w_{3}$		
	Moran’s I	*Z*-Value	*p*-Value	Moran’s I	*Z*-Value	*p*-Value	Moran’s I	*Z*-Value	*p*-Value
2010	0.244	2.931	0.003	0.203	1.940	0.052	0.780	6.167	0.000
2011	0.265	3.154	0.002	0.249	2.320	0.020	0.763	6.056	0.000
2012	0.259	3.075	0.002	0.254	2.343	0.019	0.747	5.893	0.000
2013	0.251	2.984	0.003	0.236	2.198	0.028	0.738	5.823	0.000
2014	0.258	3.074	0.002	0.267	2.455	0.014	0.729	5.772	0.000
2015	0.281	3.295	0.001	0.291	2.638	0.008	0.710	5.598	0.000
2016	0.281	3.283	0.001	0.294	2.655	0.008	0.718	5.654	0.000
2017	0.271	3.185	0.001	0.275	2.508	0.012	0.720	5.668	0.000

**Table 3:** Results of LM tests.

Test	Statistic	df	*p*-Value
LM-error	2.773	1	0.096 *
Robust LM-error	2.893	1	0.089 *
LM-lag	0.025	1	0.875
Robust LM-lag	0.145	1	0.703

The result of *er*_1_ under *w*_1_ matrix, * denote significance at the 0.1 levels.

**Table 4:** Results of Hausman test.

SAR	SEM	SDM
chi2(8) = (b−B)’ [(V_b−V_B)${}^{\wedge }$(−	chi2(7) = (b−B)’[(V_b−V_B)${}^{\wedge }$(−	chi2(8) = (b−B)’[(V_b−V_B)${}^{\wedge }$(−
1)](b−B) = −326.26	1)](b−B) = −29.10	1)](b−B) = −112.41
chi2 < 0.0000	chi2 < 0.0000	chi2 < 0.0000

The result of *er*_1_ under *w*_1_ matrix.

**Table 5:** Comparison of SDM and SEM under $w_{1}$ weight matrix.

VARIABLES	SDM				SEM			
	${\mathrm{er}}_{1}$	${\mathrm{er}}_{1}$	${\mathrm{er}}_{2}$	${\mathrm{er}}_{2}$	${\mathrm{er}}_{1}$	${\mathrm{er}}_{1}$	${\mathrm{er}}_{2}$	${\mathrm{er}}_{2}$
	(1)	(2)	(3)	(4)	(5)	(6)	(7)	(8)
gf	1.045 *** (3.07)	1.242 *** (3.52)	0.687 * (1.88)	0.868 ** (2.29)	0.606 * (1.72)	0.740 ** (2.09)	0.561 (1.51)	0.692 * (1.82)
Wgf	3.814 *** (4.46)	4.214 *** (3.91)	2.581 *** (2.85)	2.500 ** (2.24)				
$er_{1}$ or $er_{2}$	−0.930 *** (−7.60)	−0.688 *** (−4.01)	0.066 *** (1.68)	0.067 * (1.72)	−0.634 *** (−5.75)	−0.404 ** (−2.46)	0.110 *** (2.86)	0.109 *** (2.85)
$Wer_{1}$ or $Wer_{2}$	−1.347 *** (−3.51)	−1.304 *** (−2.97)	−0.094 ** (−2.30)	−0.096 ** (−2.39)				
$gf\times er_{1}$ or $gf\times er_{2}$		4.682 ** (2.05)		−0.309 ** (−2.01)		4.412 * (1.86)		−0.236 (−1.48)
$W\left(gf\times er_{1}\right)$ or $W\left(gf\times er_{2}\right)$		1.279 (0.20)		0.274 (0.66)				
IO	2.013 (1.32)	2.051 (1.36)	3.251 * (1.95)	3.041 * (1.84)	2.817 * (1.88)	2.944 ** (1.99)	4.103 *** (2.60)	3.961 ** (2.52)
UR	1.492 ** (2.38)	1.573 ** (2.53)	2.991 *** (4.52)	2.966 *** (4.51)	1.264 ** (2.15)	1.280 ** (2.20)	1.801 *** (2.82)	1.779 *** (2.80)
TO	0.576 *** (3.23)	0.467 ** (2.37)	0.477 ** (2.33)	0.349 (1.63)	0.684 *** (3.88)	0.525 *** (2.70)	0.592 *** (3.04)	0.505 ** (2.50)
HC	1106.390 (1.29)	1199.955 (1.40)	225.303 (0.24)	535.900 (0.57)	1157.821 (1.37)	1300.240 (1.55)	848.356 (0.95)	1121.405 (1.24)
IPP	6.817 ** (2.25)	6.444 ** (2.13)	6.559 ** (1.99)	6.862 ** (2.09)	4.036 (1.39)	3.189 (1.09)	0.885 (0.29)	0.896 (0.30)
_cons	3.110 *** (4.06)	2.866 *** (3.70)	0.328 (0.55)	0.422 (0.72)	6.593 *** (16.33)	6.583 *** (16.19)	5.349 *** (9.02)	5.348 (9.04)
ρ or λ	0.266 *** (3.11)	0.273 *** (3.20)	0.407 *** (5.05)	0.402 *** (4.96)	0.908 *** (43.97)	0.992 *** (45.44)	0.908 *** (41.98)	0.909 *** (42.55)
sigma2_*e*	0.029 *** (10.10)	0.028 *** (10.04)	0.034 *** (10.07)	0.033 *** (10.05)	0.034 *** (9.94)	0.033 *** (9.89)	0.039 *** (9.89)	0.038 *** (9.88)
R−squared	0.486	0.452	0.302	0.278	0.460	0.441	0.456	0.445

Note: ***, **, * denote significance at the 0.01, 0.05, and 0.1 levels, respectively. *Z*-values are in parentheses and *p*-values are in brackets.

**Table 6:** Spatial regression results of $er_{1}$ model and $er_{2}$ model under $w_{1}$, $w_{2}$, $w_{3}$ weight matrices.

VARIABLES	${\mathrm{er}}_{1}$ Model						${\mathrm{er}}_{2}$ Model					
	(1)	(2)	(3)	(4)	(5)	(6)	(7)	(8)	(9)	(10)	(11)	(12)
	$w_{1}$	$w_{1}$	$w_{2}$	$w_{2}$	$w_{3}$	$w_{3}$	$w_{1}$	$w_{1}$	$w_{2}$	$w_{2}$	$w_{3}$	$w_{3}$
gf	1.045 *** (3.07)	1.242 *** (3.52)	1.177 *** (3.31)	1.363 *** (3.62)	0.625 ** (2.04)	0.784 ** (2.53)	0.687 * (1.88)	0.868 ** (2.29)	0.901 ** (2.40)	0.858 ** (2.20)	0.875 ** (2.15)	0.905 ** (2.17)
Wgf	3.814 *** (4.46)	4.214 *** (3.91)	0.821 (1.62)	1.017 (1.58)	−0.833 (−1.33)	−0.269 (−0.35)	2.581 *** (2.85)	2.500 ** (2.24)	−0.299 *** (−0.57)	−0.781 (−1.37)	−0.521 (−0.70)	−0.631 (−0.76)
$er_{1}$ or $er_{2}$	−0.930 *** (−7.60)	−0.688 *** (−4.01)	−0.960 *** (−7.57)	−0.693 *** (−3.98)	−0.533 *** (−6.07)	−0.204 (−1.62)	0.066 *** (1.68)	0.067 * (1.72)	0.111 *** (2.59)	0.096 ** (2.28)	0.105 *** (2.75)	0.101 *** (2.63)
$Wer_{1}$ or $Wer_{2}$	−1.347 *** (−3.51)	−1.304 *** (−2.97)	−0.441 (−1.29)	−0.492 (−1.38)	0.598 * (1.84)	0.911 *** (2.67)	−0.094 ** (−2.30)	−0.096 ** (−2.39)	−0.125 *** (−2.83)	−0.111 ** (−2.55)	−0.143 *** (−3.67)	−0.139 *** (−3.50)
$gf\;\times \;er_{1}$ or $gf\;\times \;er_{2}$		4.682 ** (2.05)		5.362 ** (2.22)		6.287 *** (3.54)		−0.309 ** (−2.01)		−0.237 (−1.51)		−0.064 (−0.43)
$W(gf\;\times \;er_{1})$ or $W(gf\;\times \;er_{2})$		1.279 (0.20)		−0.695 (−0.13)		8.654 (1.60)		0.274 (0.66)		0.712 ** (2.51)		0.135 (0.39)
IO	2.013 (1.32)	2.051 (1.36)	2.466 (1.53)	2.540 (1.59)	1.592 (1.33)	2.106 * (1.79)	3.251 * (1.95)	3.041 * (1.84)	3.735 ** (2.10)	3.576 ** (2.04)	2.470 (1.53)	2.380 (1.46)
UR	1.492 ** (2.38)	1.573 ** (2.53)	2.466 *** (3.60)	2.376 *** (3.73)	0.994 * (1.88)	1.002 ** (1.97)	2.991 *** (4.52)	2.966 *** (4.51)	3.798 *** (5.60)	3.721 *** (5.56)	3.343 *** (5.48)	3.365 *** (5.50)
TO	0.576 *** (3.23)	0.467 ** (2.37)	0.559 *** (2.96)	0.408 ** (2.01)	0.746 *** (4.60)	0.564 *** (3.46)	0.477 ** (2.33)	0.349 (1.63)	0.454 ** (2.09)	0.331 (1.49)	0.498 ** (2.40)	0.476 ** (2.23)
HC	1106.390 (1.29)	1199.955 (1.40)	767.725 (0.94)	939.662 (1.12)	−28.801 (−0.04)	64.840 (0.10)	225.303 (0.24)	535.900 (0.57)	591.282 (0.64)	885.717 (0.95)	456.128 (0.53)	446.417 (0.54)
IPP	6.817 ** (2.25)	6.444 ** (2.13)	8.373 *** (2.92)	7.984 *** (2.81)	3.712 (1.37)	2.400 *** (0.91)	6.559 ** (1.99)	6.862 ** (2.09)	5.190 * (1.65)	65.143 * (1.66)	6.845 ** (2.24)	7.023 ** (2.28)
_cons	3.110 *** (4.06)	2.866 *** (3.70)	1.528 * (1.91)	1.309 (1.64)			0.328 (0.55)	0.422 (0.72)	−0.526 (−0.84)	−0.484 (−0.78)	−0.473 (−0.96)	−0.510 (−1.02)
rho	0.266 *** (3.11)	0.273 *** (3.20)	0.455 *** (6.80)	0.458 *** (6.86)	−0.336 *** (−4.21)	−0.375 *** (−4.78)	0.407 *** (5.05)	0.402 *** (4.96)	0.531 *** (8.41)	0.521 *** (8.24)	0.302 *** (4.48)	0.298 *** (4.34)
sigma2_*e*	0.029 *** (10.10)	0.028 *** (10.04)	0.032 *** (9.97)	0.030 *** (9.92)	0.017 *** (10.77)	0.016 *** (10.74)	0.034 *** (10.07)	0.033 *** (10.05)	0.038 *** (9.93)	0.037 *** (9.92)	0.032 *** (10.19)	0.032 *** (10.19)
R−squared	0.486	0.452	0.386	0.346	0.312	0.234	0.302	0.279	0.318	0.307	0.416	0.415

Note: ***, **, * denote significance at the 0.01, 0.05, and 0.1 levels, respectively. *Z*-values are in parentheses and *p*-values are in brackets.

**Table 7:** Spatial spillover effects of $er_{1}$ model and $er_{2}$ model under $w_{1}$ weight matrix.

VARIABLES	${\mathrm{er}}_{1}$ Model			${\mathrm{er}}_{2}$ Model		
	Direct Effects	Indirect Effects	Total Effects	Direct Effects	Indirect Effects	Total Effects
gf	1.478 *** (4.09)	6.084 *** (4.64)	7.562 *** (5.36)	1.106 *** (2.82)	4.542 *** (2.82)	5.648 *** (3.27)
$er_{1}$ or $er_{2}$	−0.768 *** (−5.99)	−1.996 *** (−3.67)	−2.763 *** (−4.49)	0.060 * (1.68)	−0.109 *** (−2.67)	−0.050 ** (−2.27)
$gf\;\;\times \;er_{1}$ or $gf\;\;\times \;er_{2}$	4.945 ** (2.23)	3.939 (0.45)	8.884 (0.94)	−0.283 * (−1.86)	0.248 (0.38)	−0.035 (−0.05)
IO	1.714 (1.14)	−8.500 (−1.55)	−6.782 (−1.10)	2.645 (1.52)	−7.924 *** (−1.09)	−5.279 (−0.64)
UR	1.680 *** (2.73)	3.153 ** (2.23)	4.833 *** (2.86)	3.333 *** (5.20)	7.367 *** (4.32)	10.700 *** (5.57)
TO	0.456 ** (2.30)	−0.479 *** (−0.93)	−0.021 (−0.04)	0.329 (1.46)	−0.841 (−1.28)	−0.512 (−0.68)
HC	1280.223 (1.47)	19999.480 (0.92)	3279.704 (1.40)	628.050 (0.64)	2329.906 (0.72)	2957.956 (0.84)
IPP	6.614 ** (2.13)	6.606 (0.64)	13.220 (1.15)	8.400 ** (2.45)	32.495 *** (2.61)	40.894 *** (2.93)

Note: ***, **, * denote significance at the 0.01, 0.05, and 0.1 levels, respectively. *Z*-values are in parentheses and *p*-values are in brackets.

## Data Availability

Data is contained within the article.
